# Climate change and women’s mental health in two vulnerable communities of Bangladesh: An ethnographic study

**DOI:** 10.1371/journal.pgph.0002080

**Published:** 2024-06-27

**Authors:** Jean-Marc Goudet, Faria Binte Arif, Hasan Owais, Helal Uddin Ahmed, Valéry Ridde

**Affiliations:** 1 Ceped, Université Paris Cité, Inserm, IRD, Paris, France; 2 BRAC James P Grant School of Public Health, BRAC University, Dhaka, Bangladesh; 3 International Centre for Diarrhoeal Disease Research (icddr,b), Dhaka, Bangladesh; 4 National Institute of Mental Health and Hospital, Dhaka, Bangladesh; 5 Institut de Santé et Développement, Université Cheikh Anta Diop, Dakar, Sénégal; University of Groningen, NETHERLANDS

## Abstract

Climate change is one of the most significant challenges humanity faces in the 21st century, with its health impacts being profoundly felt in the most vulnerable countries. However, the mental health impacts of climate change, particularly concerning social inequality and gender dynamics, are less documented in the Global South. This paper focuses on the impact of climate change on women’s mental health in two vulnerable communities in Bangladesh. This study employed qualitative methods, including, in-depth interviews, and focus group discussions (FGDs). The communities were selected based on their vulnerability to climate change. A total of 80 participants were selected using snowball sampling, and 55 interviews and 6 FGDs were conducted. Women are particularly vulnerable to climate change impacts on mental health due to their gender roles and responsibilities. Responsible for taking care of their families, they have to face additional challenges due to climate change impacts, such as increased workload, food insecurity, and social insecurity when their husband migrates to the cities for jobs. Women also face social and cultural barriers, which exacerbate their vulnerability to climate change impacts on mental health. Socioeconomic and environmental determinants appear to be embedded and lead to psychological suffering in relation to social roles and gender norms. Interventions should be designed to address the specific needs and challenges faced by women in these communities. Policymakers should take a gender-sensitive approach to address the mental health impacts of climate change in these communities. This study contributes to the growing body of research on the gendered impacts of climate change with a trajectory approach and provides insights for future research in this area.

## Introduction

The ongoing climate crisis represents the greatest threat of this century to both human existence and biodiversity [1]. Climate change (CC) refers to a long-term shift in the weather patterns that can be identified due to natural processes and anthropogenic factors [2]. Since 2010, CC impacts on mental health are described as a global emergency. Different frameworks have the strength of showcasing various determinants in a cross-cutting approach supported by epidemiological evidence. However, very few highlight the socio-economic and environmental factors in their interactions and how they contribute to mental health trajectories at the individual level. Berry et al., described two main causal pathways between mental health outcomes and climate impacts [3]. Direct consequences occur after acute events and are described in terms of depression or psychological trauma [4]. Indirect consequences could be caused by the changes in ecosystems through different determinants such as economy, migration, or social structures [4]. For example, long-term changes such as chronic drought increased the distress of the rural population [5]. Additionally, the cumulative effects of multiple stressors associated with climate change can intensify existing mental health issues or lead to the emergence of new ones. Such stressors may include food and water insecurity [6–8], loss of livelihoods [9], displacement [10], and social networks [11]. Yet, a knowledge gap remains to characterize the chronic effects of slow-onset events on mental health issues [12]. Siegele defines slow-onset events as phenomena with chronic changes (increasing temperature, sea-level rise, salinization, ocean acidification, loss of biodiversity, etc.) as opposed to acute events (storms, floods, acute drought, etc.) that are not permanent and occur in a short time frame [13]. As the slow-onsets and their chronic consequences will keep rising, it is important to better understand the underlying mechanisms to provide adequate actions. Moreover, the slow onset events are disproportionally affecting the population living in the Global South as they are more dependent on natural resources such as water and crops. While women are considered at higher risk of suffering from post-traumatic stress disorder after any natural disaster [14], how climate changes affect their mental health remains underexplored in the Global South. Recent results show how climate change, and specifically extreme weather events do affect the social structure. Women’s vulnerabilities are shaped by cultural and gender norms, as well as their lower socio-economic status in society, which manifests itself in their domestic role, decision-making within the household, and it is accentuated during climatic events [15] and in the context of a changing climate [16]. Indeed, these events prompt men to leave their communities in search of income, while women are trapped behind to shoulder caregiving responsibilities and additional duties [17]. But the underlying mechanisms of action are not well-explained. Moreover, there is limited data available from countries in the Global South, which are often the most vulnerable to the impacts of climate change.

Bangladesh is one of the countries most exposed to climate events (cyclones, tidal surges, floods, periods of drought) [18], and over the past decade, acute events have become increasingly frequent and intense due to climate change [19]. Simultaneously, the nation faces slow-onset events (acidification, increasing temperature, salinization, loss of biodiversity, etc.) that have led to environmental disruptions [20,21]. More than 60% of the population lives in rural areas and is significantly affected by slow-onset events [22]. The country is now confronting a dual crisis with the emergence of a mental health crisis. Recent studies highlight the substantial mental health burden in Bangladesh, with a 2019 average prevalence of mental disorders in the population reaching 16.8% [23]. A 2020 study published showed that 20% of rural women were screened for a major depression disorder [24]. However, mental health service providing infrastructure is limited, and very few psychiatrists are available in the country, with an estimated 260 psychiatrists for the 162 million people living in the area [25]. Moreover, research in the mental health field is underfunded and women’s mental health has been identified as one of the scientific priorities in recent times [26].

The objective of this study is to explore how climate change affects the mental health of women in vulnerable communities in the Global South especially in Bangladesh.

## Material and methods

### Study setting

This study is nested within the Clim-HB project, a large public health research that focuses on health system resilience and climate change [27]. Our research took place in two vulnerable communities in Bangladesh, one in a rural area which is disaster prone and the other in an urban area chosen due to their vulnerability and exposure to climate change.

In the rural community, the local population relies on agriculture and aquaculture (fish and shrimp culture) for their livelihoods. This community, located in the southwest region of the country, is subject to both slow-onset and acute climatic events. Moreover, 20% of the population experiences a seasonal migration after the monsoon due to the lack of employment. The second setting is a slum in the capital city. The investigation primarily focused on the poorest households in the slum, who live in stilt houses built over a canal. This setting is particularly exposed to pollution and extreme heat. These field sites are part of a larger public health research project within the framework of the Clim HB project [27]. Both populations share a pattern of migration, as a large portion of the families encountered in the slum originated from coastal regions of Bangladesh and migrated for economic reasons and sometimes environmental ones. Coastal regions are subject to significant environmental transformations as well as a scarcity of jobs, which is also related to the urbanization of the country [20–22].

### Conduct of study

We conducted a qualitative research including in-depth interviews, and focus group discussions [28]. The participants were selected using snowball sampling. For our snowball sampling, we initiated the procedure by pinpointing and interviewing a handful of participants from the chosen communities after briefing community leaders about our research objectives. In the rural area, our starting point was individuals who discussed the effects on shrimp production due to climate changes and those who proactively invited us for discussions. These primary participants then directed us to other potential participants, typically other family members or neighbors. In the urban slum, initial participants were selected based on the vulnerability of their housing and its location which was prone to flooding. These initial participants then recommended other potential participants, often including their family members or neighbors. It is essential to highlight that this sampling method proved invaluable In the communities we have studied. Using the trust and existing relationships of our initial participants, we were able to tap into a wider network, providing us with diverse and comprehensive perspectives. This approach also ensured that we operated within a “milieu of mutual acquaintances”[28], thereby establishing social relationships with participants, which enhanced the depth and credibility of our findings. Participant observation allowed us to understand the lived experiences of these communities and to familiarize ourselves with the population. The primary tool for this technique was an observation guide. We took detailed notes, capturing observed behaviors, interactions, and environmental factors in our field journal. In-depth interviews were essential in exploring the social trajectories and mental health states of individuals. We employed a semi-structured interview guide, ensuring consistency across interviews while permitting respondents to convey their unique experiences. Focus group discussion (FGD) shed light on communal perceptions, collective experiences, and community-level challenges related to the impacts of climate change. For these discussions, we used a thematic guide.

Each interview and FGD were recorded. A sociologist and public health physician, led the data collection with two research assistants (FRAs), both trained in qualitative research methods. Both RAs were fluent in Bangla and English and had prior experience working in similar community settings. A public health researcher and a psychiatrist supervised and validated the various data collection guides used in the study. Adhering to the reflexive ethnography approach [29], we conducted our study within settings characterized by mutual acquaintance in each site and over an extended period. We embarked on three extended stays in the communities over a year and a half, enabling the collection of ethnographic materials from our field journals and conducted interviews or FGDs. Data saturation was achieved during the third phase of data collection, as we could explain the observed effects and discuss them with participants. In practical terms, after each in-depth interview and FGD, we held a collective debriefing. Also, at the end of each day, we dedicated several hours to elaborating on our field notes. A debriefing session was held the following morning. This consistent routine not only reinforced reflexivity but also allowed for the ongoing adaptation of our methods, ensuring the robustness and integrity of our data collection process.

To reflect the seasonal variations that could influence mental health issues, data were collected during two period covering three months in total: in March and April during the hot season and in October 2022 after the monsoon. In each site, approximately fifteen households were followed throughout both data collection periods. We conducted interviews with various household members to triangulate the data, which helped improve the reliability and quality of the information gathered. The results of our analyses were then triangulated with those from the literature to bolster the objectivity of our findings. This approach allows us to thoroughly comprehend the barriers and coping strategies at the household level and facilitates data triangulation within the family unit, ensuring a robust and quality data collection. This method allowing us to reconstruct social trajectories and assess the impact of specific environmental events on women’s mental health. Table 1 shows that the study comprised a total of 55 interviews and 6 FGD (n = 80). Only relevant data for the research question were retained for the analysis.

**Table 1:** Number of interviews and FGDs by site and season of study in Bangladesh in 2022.

	Rural area	Urban (slum)
**Hot season 2022**	**12 In-depth interviews** • 7 mothers • 2 fathers • 1 teenage girl • 1 teenage boy • 1 professional: traditional healer **3 FGD** (teenager-girls, adults, farmers)	**14 In-depth interviews** • 8 mothers • 1 father • 2 teenage girls • 2 community clinics’ professionals • 1 pharmacist
	Informal conversation and notes taken during the ID, FGD (observation and translated sentences) and informal moments (over 150 pages of notes)	
**After moonson 2022** **(autumn)**	**13 In-depth interviews** • 5 mothers • 3 fathers • 1 teenage girl • 2 teenage boys • 1 paramedic • 1 medical doctor • **2 FGD** (children & male adults)	**16 In-depth interviews** 3 community leaders • 11 mothers • 2 fathers **1 FGD** with male local leaders
	Informal conversation and notes taken during the ID, FGD (observation and translated sentences) and informal moments (over 100 pages of notes)	

All the interviews were conducted in Bengali with the presence of a Bengali-English translator, except for one interview which was directly conducted in English with a health professional. During the second fieldwork, the researchers returned to the same individuals to conduct a follow-up and refine the data obtained from the first fieldwork. In each site, around fifteen families were interviewed. When possible, researchers conducted interviews with different household members, to triangulate and gain a comprehensive understanding of the family’s situation. The audio recordings were transcribed by a native speaker. Observational data were compiled by a sociologist and public health physician after discussion with FRAs to ensure accurate social and cultural comprehension. First, we triangulated data for each respondent to validate key information. Next, we applied a data reduction strategy, focusing on material specifically relevant to our research question. Responses from each participant were first sorted according to the posed questions. They were then further categorized by gender, age, specific setting, and based on whether participants had current or historical experiences with mental health issues. A thematic analysis was then performed based on the social determinants framework from WHO and Lund et al. [30,31]. This process enabled the identification of specific themes and subthemes pertinent to our research problem. Those selected for detailed analysis were recurring patterns observed across various sources, which highlighted the intricate interplay between women’s mental health and climate change. The results section delineates the themes and subthemes identified through this thematic analysis.

### Data analysis

To understand how power relations among gender are constituted in a changing climate, it was combined to specific dimension (access to resources / division of labour within and beyond the household) from the gender framework [32]. Following this, emerging themes were identified and integrated into the framework analysis. This dual methodology enabled us to systematically pinpoint emerging themes and sub-themes, while also allowing for the revisitation and revision of our codes as our analysis unfolded.

The authors had access to non-anonymized individual participant data, but it is important to note that the data presented in this study have undergone a rigorous anonymization process and have been maintained with strict confidentiality.

## Ethics declarations

All participants provided signed consent to participate. Written consent was obtained from both the parents and the individuals themselves for participants under the age of 18, ensuring that both parties were informed and gave their consent to take part of this study. The study protocol was reviewed and approved by the ethics committee from the Institutional Review Board (IRB) of the BRAC James P Grant School of Public Health, BRAC University (ref: IRB‑19 November’20–050), nationally recognized, in Bangladesh.

## Results

**Fig 1:**
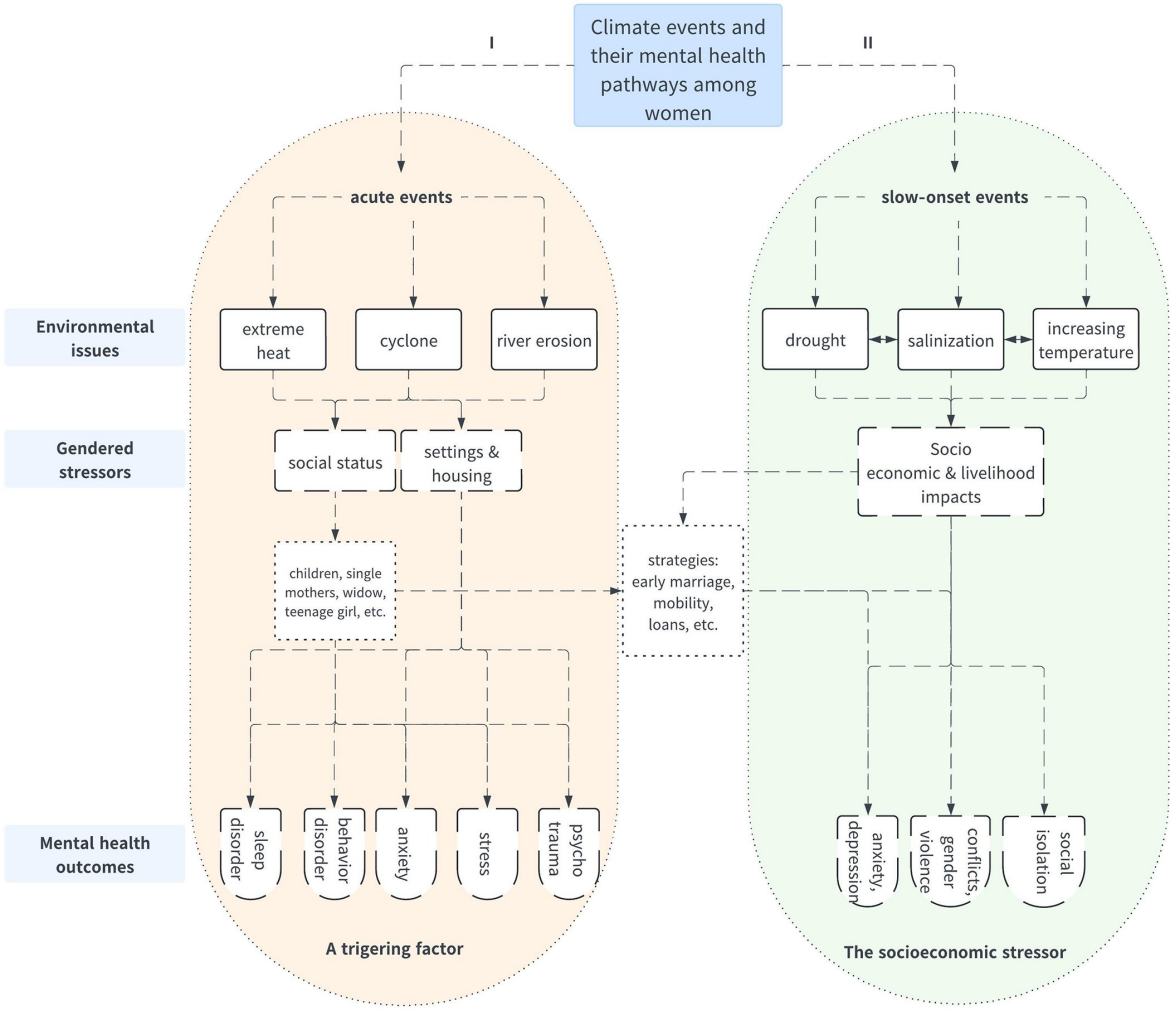
Two main causal pathways in which climate change may affect mental health of women in vulnerable communities.

**Fig 2:**
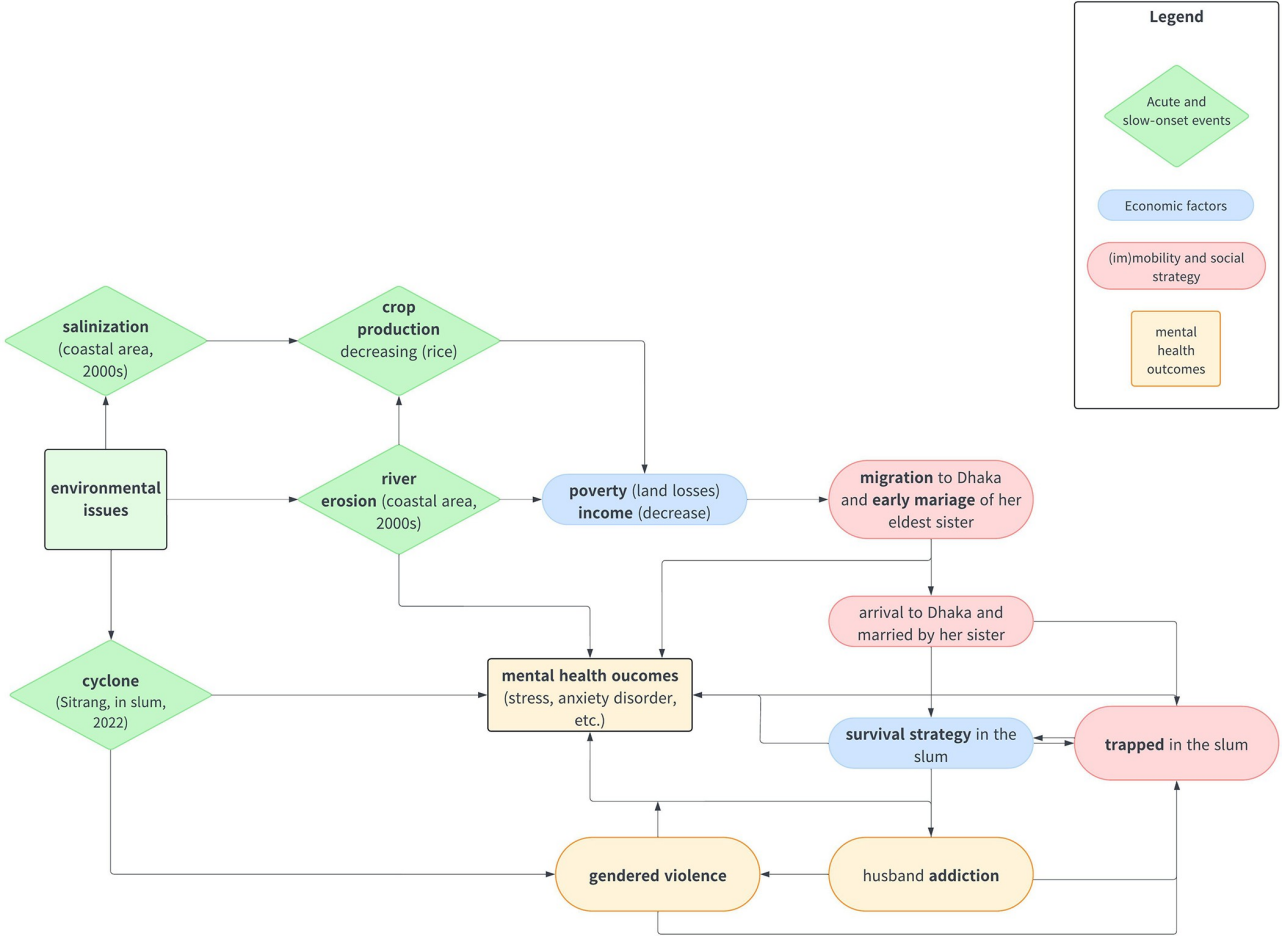
A cumulative pathway of mental health outcomes at the individual level.

Using our ethnographic approach and analyzing women’s mental health trajectories, we identified two principal pathways by which climate change influences mental health outcomes along gender lines in these vulnerable communities. These two pathways are found regardless of whether the setting is rural or urban and appear to be interconnected, depending on the social and environmental context; they often manifest as cumulative effects that shape women’s mental health trajectories. The Fig 1 presents the two main pathways through which climate change can affect the mental health of mothers and children. The diagram is based on Berry et al.’s framework, which illustrates how climate impacts on mental health occur in two ways: directly in the case of extreme events and indirectly through livelihood impacts, notably. We aim to demonstrate how this manifests through individual trajectories, highlighting the pathways taken to illustrate the importance of the interaction between socio-economic and environmental determinants. It is not exhaustive, as it does not list all environmental issues and stressors or mental health outcomes, but it provides a foundational explanation that elucidates the differences found in our data analysis. It highlights social resources and strategies and should be understood dynamically, with an interaction between the two pathways at the individual level (see Fig 2).

In presenting the results of our analysis, we will first discuss the "socioeconomic stressor" pathway followed by the "triggering factor" pathway. We will analyze each pathway through subsections that cover stressors, resources, and community vulnerabilities to understand how this shape individual mental health. The roles of gender, social status, and economic opportunities then become crucial.

### Socio-economic stressor as an intermediate factor

This first pathway contributes to exacerbating pre-existing gender inequalities arising from economic determinants. It leads to anxiety and depressive-like disorders by amplifying individual vulnerabilities through the impact on livelihood.

#### The burden of motherhood: Navigating responsibilities and the debt cycle

Climate change directly impacts the livelihoods of the most vulnerable populations. Due to drought, people from rural setting are experiencing a decline in agricultural production and shrimp yield. The immediate effect on market prices leads to food insecurity that challenges mothers’ responsibilities. It results in increased anxiety for them and potential adverse effects on the physical health of their children. This affects all families in the studied village, including those with a higher socio-economic status.

*Mother 1: There are some problems*. *Suppose*, *It has become like this due to the price hike*.

Interviewer (I): Any problems to feed for the children?

*Mother 1*:*In that situation*, *even if we are starving*, *we have to feed them*. *We have to meet their need*.

To meet the daily needs of the family members, they seek small loans. This suffering is amplified in households that are already in economic difficulty with debt.

I: You explained this year it did not rain much and it rained heavily for only 2 or 3 days. Do you think you have faced any problems with the lack of rainfall?

*Mother 2*: *We have faced some problems with the lack of rainfall such as we had to irrigate our land manually*, *and we have faced problems regarding jute retting (…) also with the money lender*. *So*, *we had to take money from our relatives as well*. *It was very stressful*.

Those that are in-debt experience the situation as one of the most difficult in their lives.

The situation seems worse for them due to a reduced ability to implement coping strategies. Indeed, the mother 1, whose husband belongs to a higher social class and has attempted to engage in local politics, holds significant social and symbolic capital in the village. The family has better economic resources and greater diversification. They own a pond with fish, a small vegetable garden, and a chicken coop. In the opposite, mother 2 and her husband have a small land and a little grocery store that experienced the COVID crisis, and they both come from impoverished families. Furthermore, mother 2 has two young children, one of whom she is breastfeeding. When asked about her sleep quality, she expresses difficulties regarding feeding issues, the child’s crying, and nutritional problems to her child which directly impact the sleep cycle of the mother.

*Mother 2: The child annoys me a lot*. *I can’t breastfeed her properly* (…) *I could not sleep previously because of the financial crisis and now I cannot sleep properly because my child cries at night*.

I: Do you wake up a lot during the night?

*Mother 2*: *He wakes up 2 or 3 times at night*. *(…) He does not like to eat rice*. *He always asks for good food like fish*, *and eggs*.

Thus, the effects of climate change tend to follow the socio-economic gradient and the preexistent social inequities. For mother 2, who belongs to a lower class and is heavily in debt, food insecurity leads to disruptions in breastfeeding and undermines her mental health as a caregiver. On the other hand, for mother 1, the psychological distress is less severe due to her financial capacity to cope with the situation. Due to hampering in the breastfeeding leads the nutrients lacking in her child also. The impacts are continuing in the cyclic pattern.

In each instance, these situations were described by the women who bear the responsibility of caring for and feeding the children, while the interviewed men explained their strategies for obtaining income without ever mentioning the mothers’ and children’s circumstances. This indicates the overlooking tendencies of the women’s burden also be discussable.

Similarly, their economic dependence on their husbands sometimes prevents them from leaving their marriage when they consider them unfit to fulfill their roles. This dynamic is a complex interplay of maternal love, the sense of duty as a mother, and economic necessity, which together reinforce their role as the principal providers for their children’s upbringing.

*Mother 3: But after marriage*, *my husband once left his job and started pulling a rickshaw*. *He used to pull the rickshaw one day and sit at home for three days without earning any money*. *At that time*, *I got a feeling of leaving this family and staying separate from my husband*. *But I couldn’t do that because of my children*. *I was worried for them*.

Income-generating activities in rural areas are not very popular. Due to their limited social capital and reduced agency, women often cannot leave their husbands to seek economic security for their children unless they can receive support from other family members, such as their own parents.

#### Coping strategies in the household rely on the daughters’ & mothers’ sacrifices

Coping strategies for the economic situations are dependent on social roles within families and based on a division of labor. Both are exacerbated in a changing climate. Mothers, who are often housewives to raise and educate the children, bear the responsibility of caregiving and thus have fewer options for economic survival strategies. This specifically impacts their mental health.

One of the economic survival strategies presented by a mother from the slum involved not bringing all her children with her. She took the youngest child who was breastfeeding and needed her, while she left her eldest daughter with her mother-in-law. She tries to visit her daughter every three months but describes this situation as very painful for her.

I: How is your sleep now? Are you tensed about something?

Mother 4: I am bound to be tensed about my son or my daughter. I can’t do any work.

I: If you could tell us what are your thought about your children?

Mother 4: The mother has to suffer the pain of the son. Will anyone else suffer?

I: Can you sleep properly or you wake up from the sleep early?

*Mother 4*: *I do wake up suddenly*. *I think what I did to deserve this*.

She didn’t want to move to the slum but had to as her husband has job there. They are both from the coastal area where it became hard to survive. Economic migration was thought as a survival strategy decided by the husband.

Furthermore, this economic migration desired by her husband isolates her. Being far from her mother and sister, makes it even more difficult for her to cope with the situation. Besides, her inability to work as she has to take care of her son reinforce her feeling of isolation.

*Mother 4: I have to think about the rent*, *food and everything*. *But in village*, *I can share everything with my mother and my sister*, *which is very much better than this*.

Coping strategies such as daughter’s marriage or economic migration are influenced by gender and social roles, and disproportionately affect girls and women. Additionally, she explained that she had to accept being married to financially relieve her parents and could not pursue her studies.

*Mother 4: I have five siblings and I am the eldest among the sisters*. *I studied till class 6 and then my parents married me off to reduce the burden (…)*.

Economic strategies for these poor rural families are mainly decided by men so they can provide for their families, even though this becomes increasingly complicated during times of economic and environmental crisis.

In some cases, environmental events shape the economic strategies of the families.

Another participant explained, that after an environmental event, her father decided to marry her.

*Mother 5: When I was a child*, *I observed a lot of cyclones and storms in that area*.

I: Did your house suffer damage during those cyclones?

Mother 5: We had 2 houses, one of them destroyed due to cyclone It happened before the river erosion. And we lost everything including houses in the river erosion.

I: When did this river erosion happen?

Mother 5: It happened around the mid-2000’s. Before my marriage.

I: Did you got married for this reason or there were other problems in your family?

Mother 5: Before the river erosion, my family had enough land and we were financially solvent but after the incident poverty hit us. And as we were so many siblings, my father took the decision about my marriage at that time.

I: What type of land were those?

Mother 5: They were agricultural land. We used to grow rice and other crops on them.

I: Can you tell me the size of those lands?

*Mother 5*: *It was a good amount of land*. *But the whole area was collapsed in the river erosion*.

In this case, the environmental event leads to a descendant social mobility of the family. Now living in the slum, this mother face economic constrained that generate anxiety. She is concerned about her children education and a broader vision of their future.

*Mother 5: It will be much more reliable for us to survive if my husband gets a new job*. *We pay 37 USD as rent for one room and*, *my husband and my children live with me in that one room*.

I: Can you tell me about your food expenses?

*Mother 5*: *Every month it costs about 90 to 110 USD for food and children’s education*. *The house rent is separate*. (…) *I’m having some stress about thinking of the future of my children*. *Since my husband has no job*, *I’m concerned about how we will manage the finances of the upcoming days*. *I’m having headaches because of this stress*.

#### Gender-based violence

Although such circumstances have been reported even in the absence of a climate crisis [33], it has been observed that gender-based violence tends to become more severe and frequent, affecting household livelihoods and reducing the opportunities for men to generate sufficient income to meet the family’s needs, sometimes exacerbated by the climate crisis. Indeed, studies highlight an increase in gender violence or intimate partner violence in the context of climate crises and extreme events such as heatwaves, floods or droughts [34–36].

For example, the previous woman who was married after a river erosion and came in the slum faced domestic violence from her unemployed and drug-addicted husband. The reason behind this violence financial hurdles in their livelihood.

*Mother 5: Now*,*I feel stressed*, *but the reason is different*. *My husband has no work and he is addicted*, *because of that we have conflicts in the house and also many other domestic problems*, *that’s why I have stress and tension all the time*. *(…) As he used to spend the money on drugs and I tried to convince him*, *for that reason he used to beat me*. *(mother 5)*

Like other participants, she is particularly isolated, which increases her stress. Her mother-in-law is her close neighbor, support her son, and would report to him any of her complaints. This exacerbates the situation and further contributes to the respondent’s stress and feelings of isolation.

*Mother 5: I’m trying to convince my mother-in-law as well*, *but every time they say that he will quit his addiction soon but it’s not happening and these types of conversations are making conflicts between my husband and his mother and also me*. *because I’m informing my mother-in-law about my husband’s addiction and somehow*, *he is also getting to know that I informed his mother*. *That’s why there are conflicts between us also*.

Several women explained to us that when they discuss household budget management with their husbands, particularly in situations of debt, the husbands reprimand them and may physically abuse them.

*Mother 6: I used to have arguments with him*, *regarding the bazaar he used to take money from people*, *and if I ask questions about it then he was beating me*

I: He is an alcoholic?

Mother 6: No. He used to gossip spend time with his friends and spend money, this is his habit, but our house condition is not good. That’s the reason

I: When you used to say something that time he used to beat?

Mother 6: I said that, we have children, and they can’t live without eating, he didn’t do the bazaar properly.

I: He used to take loans?

*Mother 6*: *Yes*. *He used to take loans from people but he didn’t go to work*, *if I ask anything then he used to beat me*.

In the rural area, a family facing over-indebtedness also experienced the negative effects of climate change on their mental health. The woman explained that their financial situation was already poor before, but it worsened this year due to a lack of rain. The combination of financial stress and the impacts of climate change can create a challenging situation for families, particularly for women, mothers, and widows who bear the responsibility of caregiving.

*Mother 7: We also had problems with the money lender*. *So*, *we had to take money from our relatives as well*. *It was very stressful*.

I: Do you sleep well at night?

*Mother 7*: *No*, *I could not sleep previously because of the financial crisis and now I cannot sleep properly because my child cries at night*. (…)

I: How is your relationship with your husband?

Mother 7: The relationship is good but we get into fights for financial matters. People come over to ask for money and get into fights over it.

I: Does he physically abuse you along with verbal abuse?

Mother 7: He hits me at times.

I: When was the last time he hit you?

*Mother 7*: *It starts with an argument*. *I try to stay patient*. *People tend to pass judgment as I live in my father’s house*. *People will talk*, *that is why I need to remain patient*.

This inescapable situation, which is often the result of norms and social pressures placed on women regarding the importance of marriage, can significantly impact their mental health and well-being. Women may feel trapped in their circumstances, unable to leave their marriages or seek help due to the expectations and obligations associated with their roles as wives, mothers, and caregivers.

*Mother 7: Because my elder brother is very angry*, *if he listened to that*, *my husband is shouting at me or if he is misbehaving with me*, *then he will beat him*. *That’s why I don’t*.

I: He doesn’t know that he used to beat you?

Mother 7: Yes, he knows, he tried to get him arrested several times.

I: Okay.

*Mother 7*: *But he couldn’t do it because of me*. *I said how many times people get married*, *once so let it be*, *let me stay with one person*.

#### Impacts of social norms on coping strategy

The isolation experienced by many mothers and young girls in reinforced by the social norms that disregard discussions about psychological issue. This is mechanism that is both self-controlled and encouraged by men due to the significance of reputation within these communities. Reputation is not only important for marrying off their children but also for matters of social dignity.

*Mother 8: I always think if I’m able to do this then*, *it will be better but I don’t have money*, *so how I’ll do it? My children wanted to live in a good place and to have good food*, *but I am unable to do anything*. *No one will help me*, *I didn’t share with anyone*, *my son asked me not to share because people will judge us*, *I am sharing with you guys as you’re listening otherwise*, *I don’t share with anyone*, *my son is different*, *he doesn’t share his sadness with anyone*. *He has grown up*. *So*, *he doesn’t like this*.

### Climate events as a triggering factor

The second pathway highlighted by our data concerns the triggering effect of climate events, that affects more specifically children, young girls and mothers.

#### Mothers’ mental health during the COVID-19

We had already encountered this issue with environmental event during our fieldwork in April 2022 following the COVID-19 pandemic. Some women had mentioned having suicidal thoughts during that period due to their perceived failure in fulfilling their caregiving responsibilities for their children.

*Mother 9: During the covid situation*, *I and my two children were staying with my husband*. *Whenever our children asked for food*, *we were not able to provide them with food*. *That time I had a feeling of committing suicide*.

I: Have you ever tried to commit suicide?

*Mother 9*: *Yes*, *I tried to commit suicide once in the middle of the Corona situation*. *But with the poison bottle in my hand*, *I couldn’t commit suicide anymore when I thought about my children because I was worried about who’ll look after my children after I will be gone*.

#### Extreme heat and children’s behavior

During our first field in the hot season, indoor temperatures were very high and significantly higher than outdoor temperatures. Several women reported children’s sleep problems resulting into behavioral issues during the day.

*Mother 10: He [*her son*] doesn’t want to go to school; after coming back from school*, *he lies down and complains that he feels annoyed*. *(…) We take a shower 2–3 times a day*. *Sits in the field for the wind*. *We get very unweary*, *so we keep splashing water on our bodies*.

I: How do they [*children*] cope with this?

Mother 10: They play with the muddy water and spend their day mainly in the pond.

I: Do they get annoyed or not get any sleep because of the hot weather?

*Mother 10*: *They hardly sleep*.

After our recorded discussion, she explained that she could not control her child’s behavior, which led her into a negative spiral of increasing anxiety and stress, due to sleep deprivation, compounded by her child’s difficult-to-regulate behavior.

#### Environmental and specific setting

The specificities of climate events are important to consider for the policy maker and to help the most vulnerable people. Usually, the main characteristics retained are the duration, frequency, intensity of an event, geographical location such as coastal areas [37].

During our study, the cyclone Sitrang occurred and was considered by local authorities as having caused much damage due to its low intensity. However, the topography of the part of the slum involved in our study, whose houses are located on a canal, were highly affected.

For the respondent, this cyclone that occurred in October 2022 was much more difficult than one of the last cyclones recognized as having caused the most damage in Bangladesh (Amphan in 2020), even if she was at this time living in the coastal area. Indeed, here she did not receive any assistance or rescue and was trapped in her room, although she took refuges during Amphan.

*Mother 11: I think this cyclone was more dangerous*. *I stayed here the whole night*. *Yes*, *it was more dangerous for me (…) there we suffered from damage*, *one of our big trees was fallen into our house*, *our house was broken because of that*, *later we were unable to fix it*, *the government helped us*, *and send some dry food*.

The inability to access shelters for these impoverished families has made them more vulnerable, even though they have migrated from coastal areas that have been hit by multiple cyclones, but where a refuge had been accessible.

#### Housing vulnerability in the face of climate events

This importance of housing was highlighted during the Cyclone Sitrang which occurred in October 2022 and caused significant damage to families living in the slum.

Some of our respondents described the alarming situation for living in the tin houses located on the part of the slum built on stilts above a canal. During the cyclone, the rainwater arrived both from clouds and canal, whose water level began to rise suddenly in the evening.

While the majority of family took refuge in a public building, this one found trapped in her room.

*Mother 12: During the cyclone*, *our house got flooded*. *The water level was very high because our house was one of the low-level houses in this area*. *We lost many small household things such as dishes and other belongings during that flood*. *The water level rose and flooded our house at 11 pm night*. *The rain started early in the morning*. *We were sleeping in the house when the water got into our house*. *We didn’t expect that the water level would rise that much because previously when it rained very little amount of water used got into our house but that night the water level was very high*.

The situation was catastrophic as the family faced the prospect of death. While the mother recounts the extreme tension, her children were traumatized, and her daughter remains in shock several days after the event.

*Mother 12: When we were standing in bed*, *I was very tense*. *We were afraid that if the rain continued*, *the water will increase*, *and we will be stuck here and die*. *One person came during that time and he was asking if someone was stuck here in these houses*. *We shouted yes and said that we were there*. *But I don’t know whether he listened to us or not because he didn’t respond*. *Also*, *there were so many mosquitoes biting us continuously*, *so we also asked that guy if he could bring us a mosquito coil*. *But he didn’t respond*. *And no one came to rescue us later on*.

I: How were the babies doing during that time?

*Mother 12*: *We removed the sewing machine from the table and placed the table on the bed*. *Then we kept our children on top of that table*. *They sat there the whole night*. *My daughter cried the whole night because she was very afraid of seeing the water*. *She is still sick*. *My son also got sick and was afraid*, *so I took him to maulana* [the religious leader of the community], *and now he’s feeling good*. *But my daughter is still afraid and sick*.

Housing is one of the most important factors in resilience or vulnerability to climate events (heat waves, floods or cyclones). In both rural and urban sites, the houses that were less exposed to flooding and more resilient to extreme weather conditions offered greater security and protected mental health outcomes. Here too, gender plays a significant role in the inheritance that is passed down to children, reflecting an inequality in the distribution of material resources. The previous respondent, who is abused by her husband, lives in a tin house, while her brother, who wants to help her, resides in their parents’ hard-building house. She is thus doubly vulnerable as she does not live in durable housing and she does not move because her husband is not a decent enough person for him to live with his family.

I: Why don’t you stay there?

*Mother 12*: *I don’t stay there as my husband is not good*. *Maybe he can say a bad word*, *they can hear that*. *It’s not good to stay with the relative*

#### From family upheaval to climatic events: The trials of a single mother

One of the most pronounced vulnerabilities to climate events is being a single mother raising her children. One of our respondents describes the alarming situation she experienced when she was caught off guard by the rising waters. Her tin-roofed room is located on a part of the slum built on stilts above a canal. The rainwater arrived both from the clouds and from the canal, whose water level began to rise suddenly in the evening.

*Mother 13: Everyone went there in the shelter*, *in our area*, *there was so much water*, *only me*, *my sister and my child were here*, *my sister told me that: “no one is here*, *do you want to die?”*. *Then somehow*, *I catch her and I started swimming*, *it was not the condition of walking*. *I started swimming*, *carrying my child on my shoulder*, *then my sister even got fallen due to the water pressure*. *So*, *I came back to this room*.

She didn’t go in a shelter as others because she perceived it a risk as a single mother. Her brother also stayed in a tin house during the event.

*Mother 13: We were only sitting on the bed*, *but we were unable to sleep*.

I: Only you and your son?

Mother 13: Yes. Many people went from here, but I didn’t go as I don’t have any place to go, where I’ll go.

I: During the night water level was increased, you did not do anything?

*Mother 13*: *Where I will go along with a child*, *so I didn’t go outside*, *I stayed here*. *I didn’t feel good to go any other place with my children*, *and it was hard for me to go anywhere to take the shelter as a single mother*, *so that is why I didn’t take the risk*, *I stay here on the bed only*.

She was also afraid by the current situation of water in the road and her inability to protect her son from the water.

I: As everyone leaves here, no one asks you to go with them?

Mother 13: Yes, everyone was telling me to go from here, I said no, I’ll not go, I had the courage, and I said that, if my death is written here then I’ll die here along with my son, I’ll not go anywhere.

I: Why you did not go anywhere?

*Mother 13*: *I didn’t go*, *because I didn’t feel good*, *I had the courage in my mind that nothing will happen*, *if my death is written here then I’ll die here*, *if I go there*, *I can also die there*. *I had the courage in my mind*.

Finally, she left her son with her uncle who stayed as the responsible person of the tong houses. Because her son was crying too much in her arms, as she was too stressed by the situation. Consequently, she stayed alone in the room, feeling very anxious, as she constantly thought about her son and found solers in her prayers.

*Mother 13: I am a million times thankful to Almighty Allah*. *Later on*, *the next day*, *the water level decreased and the rain almost stopped*. *So*, *as it was not raining*, *I was less tense*. *(…) I was so afraid when the water level was increasing*, *and I thought maybe it was going to be the end*, *but I performed special prayers*, *and I prayed to Almighty Allah*, *to save us and also*, *and I was also remembering my Almighty Allah’s name again and again*, *and then I saw in the morning the water level was decreasing*. *Around 3–4 AM it started to decrease*, *at that time I was like the water level would not increase*, *and at that time I was having less stress*.

Her initial decision to stay alone in the tin house with her son, awaiting death, is linked to her pre-existing mental condition. This woman has experienced several depressive episodes, including one a few months before the cyclone, which left her bedridden for a month, preventing her from working and taking care of her son, who stayed with his uncle. Her psychological difficulties began during childhood, without her recalling the reason, and then following her marriage and upon discovering that she was her husband’s second wife. A few years later, her parents passed away, and her husband died accidentally, causing her pain from the memories of these lost loved ones and in relation to her own condition.

*Mother 13: Sometimes I think about my parents*, *so I feel restless*, *that time it feels like I’m not able to take a breath*, *it feels like I’m not breathing*, *once I got senseless*, *I was very sick*, *my sister got to know about it*, *and she came and she took me to the hospital*

I: apart from your parent’s incident, is there anything else that makes you feel restless?

*Mother 13*: *About my husband*, *the death of my husband*, *I think about it a lot*, *as we stayed together*, *now he is no more*, *I can also die*, *first my parents died*, *I was living with my parents*, *they died*, *then I was living with my husband*, *but he also died*, *I can also die*. *(…) Nowadays I am always thinking about my after-death situation*.

#### Isolated woman

Another woman, a widow who work as a maid and lives in a tin house, explained how she was surprised when the water level raised suddenly during cyclone Sitrang and described the stress she experienced due to the situation.

*Mother 14: Yes*, *I was afraid*, *suddenly waterlogged inside the house and also inside the road*, *there was no one here*, *only 3–4 people were here whose stuff was here*, *I wasn’t able to organize anything*.

The respondent was very tensed with exacerbated symptoms due to rumors in the slum about the occurrence of a new cyclone following Sitrang.

I: Still, you’re afraid because of this?

Mother 14: Yes, people are saying that another cyclone is coming!

I: So, you are feeling afraid?

*Mother 14*: *Yes*, *I’m feeling afraid*. *I’m not going to work for 3–4 days*. *I can’t sleep*, *my body got wet*, *everything got wet*. *I got scared of seeing the water level*, *this is a problem*!

#### Rumors

Another respondent also exhibits symptoms of anxiety and has difficulty sleeping at night due to the fear of new cyclones. This fear and anticipation of potential disasters can exacerbate mental health issues, making it difficult for individuals to focus on their daily tasks and responsibilities.

*Mother 15*: *Sometimes at midnight*, *like in the night-time*, *I feel scared*, *like maybe suddenly it can come*, *because I’m staying in the tong house because nowadays people are saying a new one is coming*, *so that’s why I’m afraid if it happened again then what I’ll do*.

### Pathways of cumulative effects on mental health: A case study

The following case is that of a woman who represents a typical example of the cumulative effects of environmental, economic, and negative feedback loops when climate events occur on the mental health. The negative feedback loop typically intertwines with economic strategies related to the household size, such as migration and early marriage, or in some cases, deciding which children will migrate to the city or stay in the village, and eventually planning to return home.

**Trajectory of a woman from the coastal area and feeling trapped in the slum**

This woman was born in a coastal area of Bangladesh, exposed to risks associated with climate events such as cyclones, river erosion, and slow-onset events (salinization). She belonged to a family of farmers who owned multiple lands for rice crops and vegetables and had two houses. During her childhood, she witnessed numerous cyclones and storms. In the early 2000s, a cyclone destroyed one of her family’s houses, but no one was injured. She occasionally suffered from nightmares involving storms destroying everything, especially when her area was flooded and waterlogged. Since the road was reconstructed and reduced waterlogging, she no longer suffers from nightmares.

In the mid-2000s, her family fell into poverty after river erosion destroyed their lands and houses. With six children, her father decided, for economic reasons, to marry his two older daughters to men from the capital, hoping for better living conditions. Her older sister moved to Dhaka and married a man from the slum through her uncle’s intervention. Later, her sister brought her to Dhaka and married her to her husband.

After her marriage, she discovered that her husband was addicted to marijuana and alcohol, spending significant amounts of money on drugs. Her husband worked as a rickshaw driver, earning about 5 USD every day. However, he sold his rickshaw six days before our visit. The couple has many expenses, including education fees for their two children in primary school (around 3 USD every month). They live in a semi-built house in the slum near the lake, paying an expensive rent of around 36 USD for one room. Food expenses for the whole family amount to around 90–110 USD.

Her husband’s drug addiction created conflicts within their marriage, and he often physically abused her. Her mother-in-law, aware of the situation, protected her son, leaving the woman feeling isolated. The Cyclone Sitrang exacerbated her stress, and she expressed "feeling very stressed with tension all the time" about her children’s future.

This case shows how the woman’s trajectory has been influenced by climate events, her family’s economic problems, and the decisions made to cope with these challenges, such as migration and early marriages. Moreover, it highlights how persistent issues, such as poverty, addiction, and family conflicts, can worsen a woman’s mental health situation, especially when she feels trapped and isolated. This case also demonstrates that women are often the most affected by the consequences of climate events and economic decisions made within their households.

Other respondents mentioned after the cyclone Sitrang their sadness about leaving a child in the village as a survival strategy in order to move to the city, only to realize they had fallen into a dead-end trap. These climatic events reawaken past pains and impact mental health by highlighting how their strategies have ultimately turned against them.

The lack of opportunity impacts all members of these communities, but it appears to be especially pronounced among women who shoulder caregiving responsibility, have limited access to education, and occupy the lowest positions within family hierarchies. Both acute and slow-onset events distinctly affect the mental health of women, mothers, and widows, as it is influenced by the interplay of socioeconomic factors, social networks, and environmental conditions. These intersecting factors intensify the vulnerabilities these women experience, making it even more difficult for them to cope with the consequences of climate-related events.

We emphasize the cumulative effects and the dynamics of both causal pathways (see Fig 1) to illustrate how they are reflected in a woman’s life trajectory, particularly as demonstrated in this case study. The Fig 2 portrays what we call a cumulative pathway at the individual level, to highlight what is often missing from the general framework: the interweaving of social, economic, and environmental factors throughout a woman’s life trajectory. These embedded factors are directly linked to her mental health outcomes, manifesting as anxiety and stress that are personal and specific to her experiences.

Fig 2 highlights the causal pathways where environmental events can act directly as precipitating factors of mental health outcomes or indirectly through socio-economic factors. At the individual level, rural or urban settings shaped trajectories but do not stand as independent factors due to the strategy of (im)mobility. Like Fig 1, this diagram illustrates the trajectory of our case study, yet it does not represent all possible scenarios. Nevertheless, based on a longitudinal perspective, the cumulative pathway emerges as a recurring mechanism within our data, enabling the analysis of interactions and the concomitant effects of a variety of factors on the mental health of our respondents.

## Discussion

Specifically, the challenge is to demonstrate the dynamics of interactions at the individual level between collective socio-economic and environmental factors. This helps to complement frameworks that are most often based on epidemiological results, contributing to explanatory hypotheses. Our study sought to contribute to the overlooked aspect of the literature on how climate-related mental disorders are shaped throughout life in vulnerable communities in the Global South. The chosen approach is to demonstrate how women are disproportionately affected through two main mechanisms (see Fig 1): socioeconomic stressors and acute environmental events, that may be illustrated by the cumulative pathway (Fig 2) at the individual level. Socioeconomic stressors, particularly during slow-onset environmental transformations, highlight the intersection between economic and environmental dimensions. Acute events directly cause psychological distress. The pathway, directly and indirectly related to climate change is well-established in the literature [3]. Our findings regarding acute events such as heat, and drought align with other studies that identify these events as mental health stressors with the potential to cause chronic stress [5,38–41].

In terms of causal mechanism, our results suggest that gender-related social status and roles shape individual trajectories in the context of climate change. They are key factors also found in other studies in the Global South [42]. Socioeconomic stressors appeared to be an intermediate factor. We specifically highlight that in these communities, economic survival strategies are based on the involvement of women in serving the household, which makes women’s health even more vulnerable during climate and environmental events. This exacerbates gender inequities and jeopardizes their mental health. Some studies have shown how gender violence is linked to climate events [42]. More specifically, in our research it appears that slow-onset events, exacerbated by their impact on livelihoods lead to household conflicts such as gender violence. This finding warrants further exploration and more generally we advocate for more studies conducted in the Global South and in the mid- long-term not only in the aftermath of extreme events. More broadly, our findings add to what has been established in terms of pathways on an international level [43,44], by providing insight into the longitudinal and cumulative aspects at the trajectory scale (see Fig 2). An additional dimension of our ethnographic work is to show how mental disorders are linked to the social, environmental, and economic histories of communities and are incorporated at the individual level, sometimes manifesting as vulnerabilities, particularly among women in the surveyed communities.

Furthermore, our study allows for an analysis of cumulative environmental, economic, and social events by examining individual trajectories, opening up new perspectives for mental health research in the Global South. This approach allows us to identify both the structural sociocultural factors and the individual psychological factors that shape a person’s mental health trajectory. It aligns with recent attempts to describe the causal process of mental health related to climate change examining how factors interact and influence one other [39].

The specificities of climate events are important to consider for the policy maker and in order to help the most vulnerable people. Usually, the main characteristics retained are the duration, frequency, intensity of an event, geographical location such as coastal areas [37]. Our results showed the difference in perception and experiences lived by the most vulnerable for an event considered as small-scale by the general authorities. While not of importance to the general population, it greatly affected both the living and economic conditions of a vulnerable community and their mental health. It is crucial for decision-makers to anticipate the impacts based on the topographic vulnerabilities of the sites and the individuals, in order to promote the establishment of shelters and the provision of material aid, even during less intense events such as Cyclone Sitrang. This situation is encountered by both men and women; however, it adds to gender-specific vulnerabilities [45] and should be taken into account to grasp the cumulative impact of vulnerabilities on individuals’ life trajectories.

Understanding the gender-specific vulnerabilities related to climate events is important to address the cumulative impact of vulnerabilities on individuals’ life trajectories. Mental health outcomes linked to SDGs will be difficult to achieve for women if the climate issue is not taken into account as it disproportionately affects women by reinforcing their vulnerabilities linked to social structures and producing new gender inequities.

More specific studies should be made in the Global South because the indirect impacts of climate change on mental health are mostly uncovered as it happened mostly in the rural setting. Yet, these countries are more vulnerable to climate events even if they are from far less responsible of the changing climate [46].

Our results highlight the importance for future research to consider psychological trajectories while taking into account health determinants, social factors, as well as environmental factors. This approach aims to better understand the mechanisms that affect populations and those shaping their resilience when they face several challenges. Our analysis confirms the research findings regarding the floods that occurred in Nigeria in 2011. Researchers found that this event impacted the childcare of women’s activities leading to increased anxiety about the health and safety of their children [47]. We also find in accordance with the literature various determinants that affect the link between mental health and climate change among women. Notably, one of the key factors is the socioeconomic status of women [42], which can exacerbate their vulnerability to the mental health impacts of climate change. Other determinants include gender roles and expectations, gendered violence [48], limited access to resources [49] and services after natural disasters [50], education, and the disproportionate burden of caregiving responsibilities [48]. These factors can contribute to increased stress, anxiety, psychological trauma and depression among women when faced with the challenges posed by climate change [14]. We demonstrate how the combination of factors such as socioeconomic status, gender roles, limited resources, and caregiving responsibilities can create a complex web of vulnerabilities that disproportionately affect women’s mental health in the face of climate change. By examining these interconnected factors in detail, we provide a more comprehensive understanding of the gendered impacts of climate change on mental health in vulnerable communities, thereby offering valuable insights for future research and policy interventions. Further research employing a trajectory-based approach is needed to better understand the interplay of factors and their contributions to the impacts of climate change on women’s mental health in vulnerable communities.

### Limitations

Our study has limits. Our interviews were conducted in Bengali and transcribed so we might have lost a part of the information. However, the analysis was performed with the translator to overcome this risk. Our data were collected on a two-period basis, while this topic is a sensitive one and might need iterative interviews to build a greater trust from the respondents. We tried to overcome this limit by triangulating within the same household and meeting multiple time with our respondents to build a trusting relationship.

We were perceived as foreigners and outsiders which might have had an impact on the quality of data. During our initial fieldwork in the slum, we prioritized personal interviews over FGDs to foster relationships with residents. The context was challenging due to the residents’ economic struggles, limited availability, high expectations from us for immediate change, and interpersonal tensions between the residents. However, the relationship we established allowed us to conduct further individual interviews and one FGD with male local leaders during our second visit. In both settings we have been associated with their survival economic strategies and the interviews could have been biased to meet the respondent needs. The tools from the reflexive ethnography have helped us to analyze our position in each situation and how it has shaped the data.

## Conclusion

Our study underscores the heightened vulnerability of women to the mental health impacts of climate change, particularly in relation to household strategies and the direct effects of climate events. Indeed, the burden of care intensifies with heat, while droughts affecting livelihoods result in increased workload and debt. Such situations contribute significantly to anxiety and symptoms associated with depression. Two primary causal factors impacting women’s mental health emerged from our study: socioeconomic stressors and acute events. These factors, when combined with social roles and family strategy, produce cumulative effects. Each determinant can either provide support or exacerbate negative mental health outcomes for women, who are deeply involved in coping strategies amidst the disruptions caused by environmental changes.

It is crucial to consider the specific mental health impacts of climate change on vulnerable groups, such as women, mothers, and widows, particularly in rural areas where resources and support may be limited. Providing access to mental health care, financial assistance, and climate-resilient livelihoods can help alleviate the strain on these individuals and improve their overall well-being.

Addressing the specific needs and vulnerabilities of women in the context of climate change requires a multi-faceted approach that includes promoting gender equality, empowering women to make informed decisions about their lives, and providing access to resources and support networks that can help them build resilience and cope with the challenges they face. Additionally, it is crucial to engage communities and stakeholders in the process of challenging and changing harmful gender norms and expectations that contribute to the vulnerability of women in the face of climate change.

## Supporting information

S1 Checklist Inclusivity in global research. (DOCX)
